# Preparation of Magnesium Hydroxide Flame Retardant from Hydromagnesite and Enhance the Flame Retardant Performance of EVA

**DOI:** 10.3390/polym14081567

**Published:** 2022-04-12

**Authors:** Ling-Li Jiao, Peng-Cheng Zhao, Zhi-Qi Liu, Qing-Shan Wu, Dong-Qiang Yan, Yi-Lan Li, Yu-Nan Chen, Ji-Sheng Li

**Affiliations:** 1 School of Chemistry and Chemical Engineering, Anhui University, Hefei 230601, China; j2252872138@126.com (L.-L.J.); wuqingshan2021@126.com (Q.-S.W.); yandq1995@163.com (D.-Q.Y.); 2 Green Industry Innovation Research Institute, Anhui University, Hefei 230039, China; 3 School of Chemistry and Materials Science, Anhui Normal University, Wuhu 230022, China; 18055816583@163.com; 4 School of Chemical Engineering and Materials, Tianjin University of Science and Technology, Tianjin 300457, China; cyn15005515288@126.com; 5 Qinghai Institute of Salt Lakes, Chinese Academy of Sciences, Xining 810008, China; lijisheng2000@126.com

**Keywords:** hydromagnesite, ethylene-vinyl acetate, magnesium hydroxide, flame retardancy, mechanical properties

## Abstract

In this study, hydromagnesite, a rare natural hydrated alkaline magnesium carbonate, was used to synthesize magnesium hydroxide (MH) as a flame retardant for ethylene-vinyl acetate (EVA) to enhance its fire resistance and smoke suppression. Various concentrations of sodium hydroxide (NaOH) were used to alter the morphology and the flame-retardant efficiency of synthesized MH. EVA/MH composites were prepared through melt blending, and the influence of NaOH on the flame retardancy and mechanical properties was investigated by means of the limiting oxygen index (LOI), cone calorimeter test (CCT) and tensile test. The flame retardancy results demonstrated that composites exhibited remarkably improved flame retardant properties after introducing MH, reflected by an increase in the LOI value from 20% for neat EVA to roughly 38%. Additionally, the peak of heat release rate (pHRR), the total heat release (THR) and the peak of the smoke production rate for EVA3 were decreased by 37.6%, 20.7% and 44.4% compared with neat EVA, respectively. In the meantime, increasing char residues were also observed. The incorporation of different MH concentrations had a limited effect on the mechanical properties of the EVA/MH composites.

## 1. Introduction

Ethylene-vinyl acetate (EVA) copolymer with varying vinyl acetate contents is a thermoplastic polymer that has grown in popularity in recent years due to its attractive physicochemical properties [1,2]. It enjoys extensive applications in construction, transportation, electronic engineering and medicine [3,4,5]. Recently, EVA has been extensively used in the cable industry for its significant flexibility and processing properties [6]. However, EVA is extremely flammable, so it fails to meet the strict flame-resistant requirements of certain applications, such as those in the cable industry [7]. Therefore, it is necessary to improve its flame retardancy to fulfill the demand for broader applications.

Hydromagnesite (Mg_5_(CO_3_)_4_(OH)_2_·4H_2_O) [8], commonly known as “magnesia white” [9,10], is a rare natural hydrated alkaline magnesium carbonate mineral. It thermally decomposes in two stages over a temperature range between 220 °C and 550 °C, first releasing water and then carbon dioxide, leaving a solid residue of magnesium oxide [11,12]. However, hydromagnesite contains impurities such as CaCO_3_, Fe_2_O_3_ and SiO_2_, which can cause the deterioration of the properties of polymeric materials, restricting the direct utilization of hydromagnesite in polymers.

Magnesium hydroxide (MH) is one of the common environmentally friendly flame retardants that can improve flame retardancy and smoke suppression simultaneously [13]. It is popular in industrial applications owing to its low cost, high thermal stability, low toxicity and thermal isolation effect [14,15,16]. Zaghloul et al. [14] studied the effects of flame-retardant magnesium hydroxide on the mechanical properties of high-density polyethylene. The results of the experiments revealed that the reinforcement materials had notable effects on the mechanical properties of the HDPE composites with magnesium hydroxide in a weight percent range from 5 to 50 wt%. The results reported by Fan Ren et al. [17] indicated that as the MH content and mesh number increased, the flame retardant properties of MH-filled flame-retardant asphalt showed a rising trend.

Despite its extensive application, MH also shows some limitations. It was shown to easily impact the mechanical properties of composites due to the addition of high loading levels of MH fillers [18]. Therefore, in this study, the magnesium hydroxide flame retardant was prepared by hydrothermal synthesis using a calcination product of hydromagnesite from Bangor Lake, Xizang, China. The effect of magnesium hydroxide on the flame retardant properties and mechanical properties of EVA/MH composites was studied through the limiting oxygen index (LOI), cone calorimeter test (CCT) and tensile test. The fire safety performance of the composites after MH introduction was systematically evaluated.

## 2. Experimental Procedures

### 2.1. Materials

Natural hydromagnesite (4MgCO_3_·Mg(OH)_2_·4H_2_O) was obtained from Naqu of Bangor Lake in Tibet. Sodium hydroxide was purchased from Shanghai, China Titan Chemical Co., Ltd. Ethylene-vinyl acetate copolymer (EVA 265, with vinyl acetate of 28% and melt flow index of 3 g/min) was bought from Dupont Company. EVA granules were dried in an oven at 80 °C for 6 h before use. The other reagents were used as received without further treatment.

### 2.2. Synthesis of MH

Natural hydromagnesite was put into a crucible and calcined in a muffle furnace at 700 °C for 2 h.

The resulting white powder was mixed with deionized water to a slurry concentration of 10% with various concentrations of sodium hydroxide (0 mol/L, 0.5 mol/L, 1.0 mol/L, 1.5 mol/L and 2.0 mol/L) and then put into a Teflon autoclave at 140 °C for 4 h. The product was filtered and dried at 105 °C for 6 h and ground for further application.

The formulations of neat EVA and EVA/MH composites are listed in Table 1. The composites were prepared by blending EVA with different MH concentrations using a twin-screw extruder at 150 °C for 15 min at a mixing speed of 35 rpm. Afterwards, the uniformly mixed materials were laminated into two types of sheets with thicknesses of 1.6 mm and 3 mm, respectively.

### 2.3. Measurements

Thermogravimetric analysis of the sample was performed using a Labsys Evo (Perkin Elmer Instruments (Shanghai) Co.,Ltd. (US company), Shanghai, China) instrument. About 15.0 mg of sample was put into an alumina crucible and heated from ambient temperature to 800 °C. The heating rate was set to 10 °C/min under a nitrogen atmosphere (flow rate of 30 mL/min).

The crystalline phases were determined by XRD. The power of the Cu target (λ = 1.540598) was 40 kV × 35 mA, and the scanning range was 10~80°.

A scanning electron microscope (Hitachi, Tokyo, Japan) was used to examine the morphology of MH at a voltage of 2.0 kV. The specimens were previously coated with a conductive layer of gold.

Particle size distribution was measured by a Bettersize2600 laser particle size analyzer produced by Bettersize Instruments Ltd. (Dandong, China). The powder was dispersed by ultrasound at 1600 r for 3 min.

The mechanical properties were determined in accordance with ISO 527-1 using an electronic universal testing machine (suns technology Co., Ltd., Shenzhen, China); 5 specimens (115 mm × 6 mm × 1.6 mm) from each material series were used for the determination, and the tensile speed was 50 mm/min.

A ZR-01 oxygen index meter (Motis Technology Co., Ltd., Kunshan, China) was used to determine the limiting oxygen index (LOI) of specimens with dimensions of 150 mm × 6.5 mm × 1.6 mm according to ASTM D2863-77 standard.

The cone calorimeter (Motis Technology Co., Ltd., Kunshan, China) test was performed according to ISO 5660 standard procedures. Each specimen with dimensions of 100 mm × 100 mm × 3 mm was wrapped in aluminum foil and exposed horizontally to a cone shape heater with an external heat flux of 50 kW/m^2^.

## 3. Results and Discussion

### 3.1. Thermal Decomposition Behavior of Hydromagnesite

**Figure 1:**
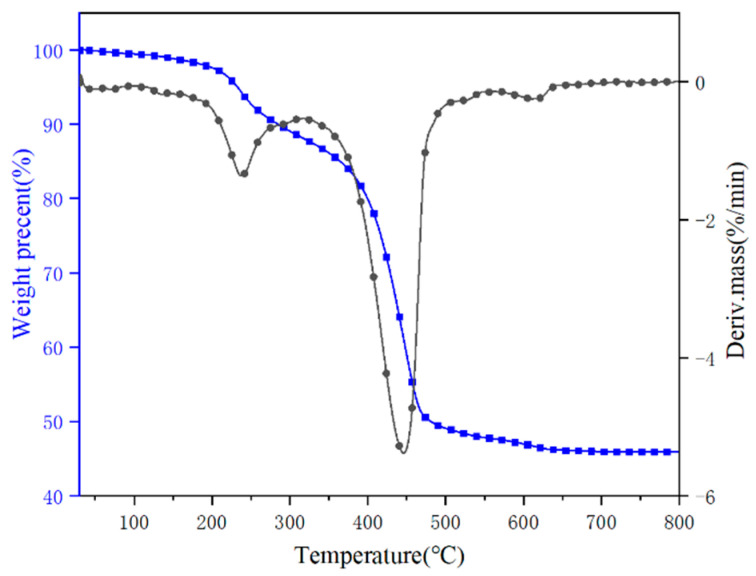
TG and DTG curves of hydromagnesite sample.

The thermal decomposition behavior of hydromagnesite was evaluated by TGA under a nitrogen atmosphere. The TG/DTG results are plotted in Figure 1, and the corresponding data are given in Table 2. The initial decomposition temperature (T_−5%_) is defined as the temperature at which 5 wt% of the mass is lost, and T_max_ is the temperature at which the maximum mass loss rate for the sample occurs. The maximum mass loss temperature of hydromagnesite (T_max1_, T_max2_ and T_max3_) for the three decomposition steps were 240, 450 and 618 °C, respectively. The sample presented a high residue of 52.82% under a nitrogen atmosphere.

The results revealed that the hydromagnesite underwent weight losses that started at 150 °C and ended at 680 °C. The decomposition process of the sample mass loss can be divided into three intervals based on the steps of mass loss: during the first interval (150~350 °C), the water of the crystal was lost by hydrothermal absorption of hydromagnesite, resulting in the initial mass loss. The chemical reaction equation is:

Mg_5_(CO_3_)_3_(OH)_2_·4H_2_O → Mg_5_(CO_3_)_3_(OH)_2_ + 4H_2_O (1)

In the second temperature interval (350~520 °C), water molecules were released again, and the hydroxyl group in the crystal structure was destroyed, releasing carbon dioxide. The chemical reaction equation is:

Mg_5_(CO_3_)_3_(OH)_2_ → 2MgCO_3_ + 3MgO + 2CO_2_ + H_2_O (2)

In the third temperature interval (520~680 °C), the thermal decomposition of the specimen released CO_2_, which was completely transformed into MgO [8,12]. The chemical reaction equation is:

2MgCO_3_ → 2MgO + 2CO_2_ (3)

The mass losses in each stage were 12.69%, 37.65% and 2.48%, respectively.

### 3.2. Morphological Characterization of MH

**Figure 2:**
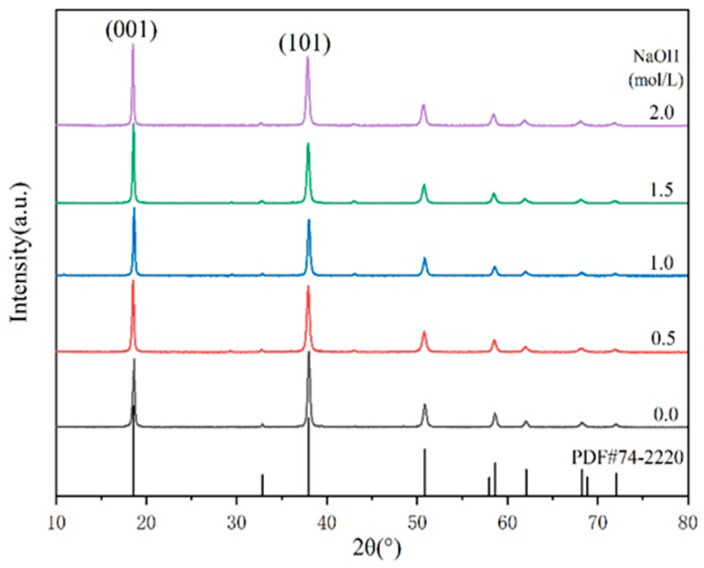
XRD patterns of different MH powders.

Figure 2 presents the correlation between the concentration of sodium hydroxide and the properties of MH samples. The average particle sizes of the products after the hydrothermal reaction and the statistical data of I_(001)_/I_(101)_ are listed in Table 3.

**Figure 3:**
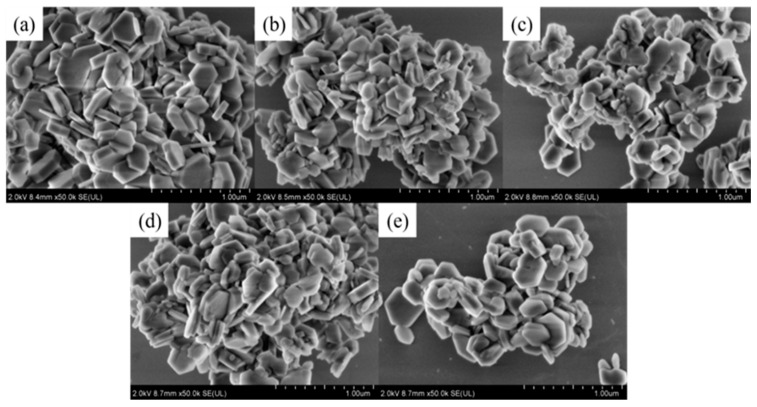
SEM micrographs of MH particles prepared with different NaOH concentrations: (**a**) 0 mol/L; (**b**) 0.5 mol/L; (**c**) 1.0 mol/L; (**d**) 1.5 mol/L; (**e**) 2.0 mol/L.

It can be seen in Table 3 that the increase in OH^−^ concentration in the hydrothermal medium led to an increase in the intensity of each diffraction peak, completely shaping the crystal and resulting in a larger grain size (Figure 3). This indicates that increasing OH^−^ concentration was beneficial to the growth of magnesium hydroxide crystallites.

**Figure 4:**
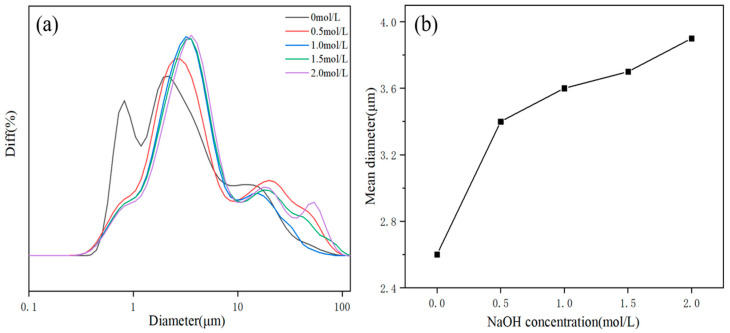
The particle size distribution diagram (**a**) and mean diameter (**b**) of MH powders. Diff, difference distribution, that is, the percentage of particles in a series of particle diameter intervals.

Figure 3 shows the effect of NaOH on the morphology of the product. As shown in Figure 3 and Table 3, with increasing OH^−^ concentration, the particle size and the intensity ratios I(001)/I(101) increased accordingly. These phenomena demonstrated that the increase in OH^−^ concentration was conducive to the growth of the magnesium hydroxide (001) crystal plane. However, the growth rate of the magnesium hydroxide (001) crystal plane was not linearly related to the OH^−^ concentration. The reason could be that the generation of MH covered the growth interface. After reaching a critical concentration, contributing to the nonequilibrium of the ratio of Mg^2+^ and OH^−^ concentration, the shielding effect dominated and prevented further growth of the crystal plane. The particle size distribution diagram and mean diameter of MH products are shown in Figure 4.

### 3.3. Thermal Stability

**Figure 5:**
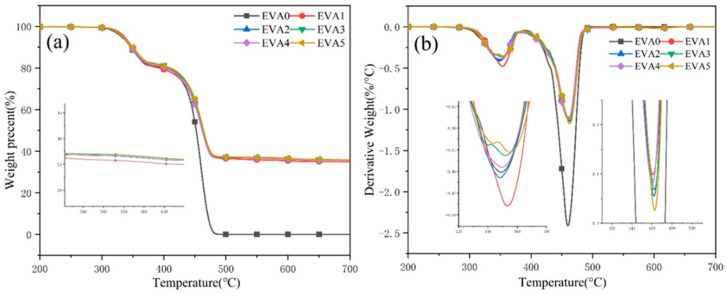
(**a**) TG and (**b**) DTG curves of neat EVA and EVA/MH composites.

The thermal stabilities of neat EVA and EVA/MH composites evaluated by TG under a nitrogen atmosphere are displayed in Figure 5 and Table 4. All of the samples followed two decomposition steps. The maximum mass loss temperatures (T_max1_ and T_max2_) of neat EVA were 348.9 and 459.9 °C. The first decomposition step was due to the loss of acetic acid, and the second involved random chain scission of the remaining material, forming unsaturated vapor species [19,20]. After the introduction of MH, the initial decomposition temperature (5% mass loss) of composites had a slight increase, which was higher than that of neat EVA (331.8 °C). Compared with neat EVA, the EVA/MH composites had higher T_max1_ and char residue yield, which demonstrates that the addition of MH improved the thermal stability of EVA, promoting carbon formation.

### 3.4. Flammability

**Figure 6:**
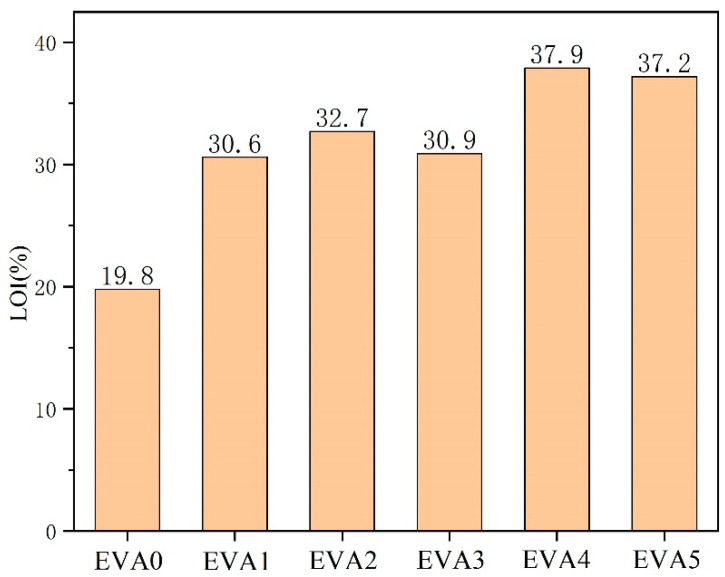
Limiting oxygen index (LOI) results of neat EVA and EVA/MH composites.

LOI tests are commonly used to assess the flammability of materials [21]. Materials are considered non-combustible when their limiting oxygen index exceeds 26% [22]. The LOI values of EVA/MH composites are shown in Figure 6. The LOI of neat EVA was only 19.8%. In comparison, EVA1, EVA2, EVA3, EVA4 and EVA5 had increased LOIs of 30.6%, 32.7%, 30.9%, 37.9% and 37.2%, respectively.

**Figure 7:**
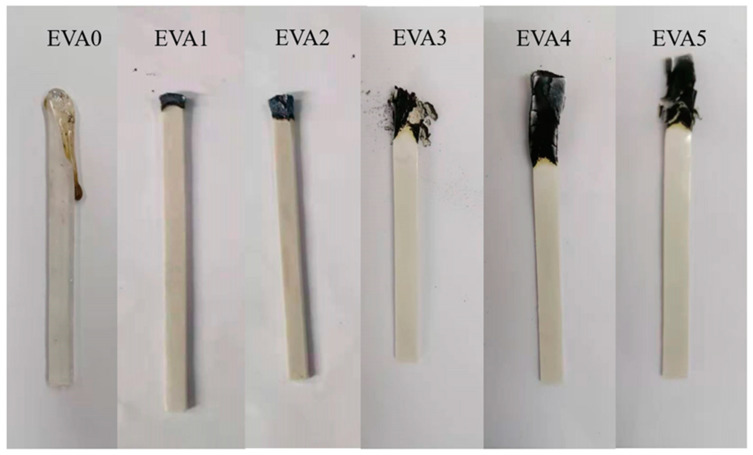
Digital photos of neat EVA and EVA/MH composites after LOI test.

Some of the digital photos of neat EVA and EVA/MH composites after the LOI test are displayed in Figure 7. The droplet phenomenon occurred during the combustion of neat EVA. There was no carbon layer on the surface of the combustion end. The addition of MH to the EVA matrix increased the LOI value, which was attributed to the barrier effect against heat and volatiles. Therefore, with the highest char yield after the test, which resulted in stronger heat dissipation and extinguished the fire, EVA4 correspondingly showed the highest LOI value.

### 3.5. Combustion Behavior

**Figure 8:**
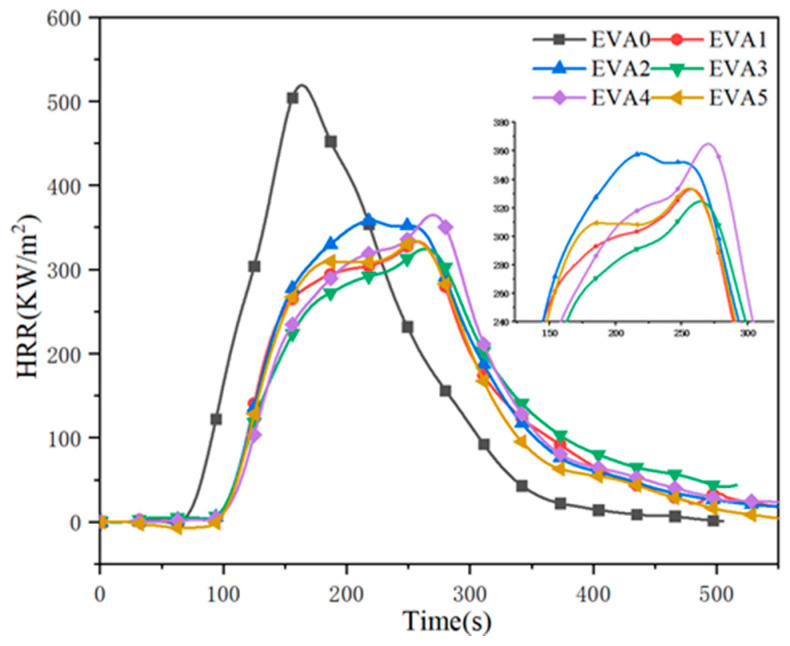
Heat release rate curves of neat EVA and EVA/MH composites.

The cone calorimeter test (CCT) is a widely used method for determining seniority and comparing the combustibility of polymeric materials [2,23,24]. Herein, the heat release rate (HRR) curves of neat EVA and EVA/MH composites are depicted in Figure 8, and the comprehensive data are summarized in Table 5. It can be seen that the peak of the HRR curve of EVA0 was higher than that of the other specimens, and the time to ignition (TTI) of EVA0 also happened earlier, which denotes a faster flame spread and major fire hazard [25,26]. Specifically, the neat EVA burnt out within 552 s, and at 100 s, the peak heat release rate (pHRR) of 559 KW/m^2^ was obtained. Compared to that, the pHRR values of the EVA/MH composites decreased significantly, which provided evidence that MH had a significant effect on reducing the fire hazard. Among the composites, EVA3 led to a decrease in HRR, which dropped to 349 kW/m^2^, and a 77 s delay in TTI. An apparent HRR plateau preceded the peak toward the end of combustion, indicating the formation of a protective char layer.

**Figure 9:**
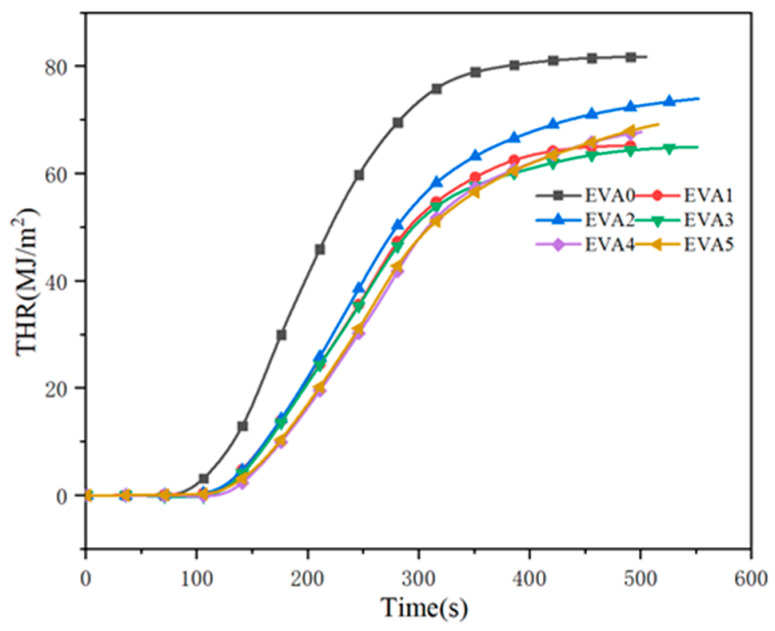
Total heat release curves of neat EVA and EVA/MH composites.

Total heat release (THR) is the total amount of heat released by material from ignition to flame extinction under a certain thermal radiation intensity [27]. The THR plots in Figure 9 show that the THR value of EVA0 was much higher than that of the other specimens. It was found that the changes in THR curves were similar to the HRR curves. For neat EVA, the value of THR began to increase at about 100 s and rapidly increased to the maximum value of 81.8 MJ/m^2^. Compared with EVA0, the THR value of EVA1 was greatly reduced, indicating that the combustion was inhibited by the addition of MH to the system. Moreover, among all of the composites, EVA3 showed the lowest THR of 65.0 MJ/m^2^.

**Figure 10:**
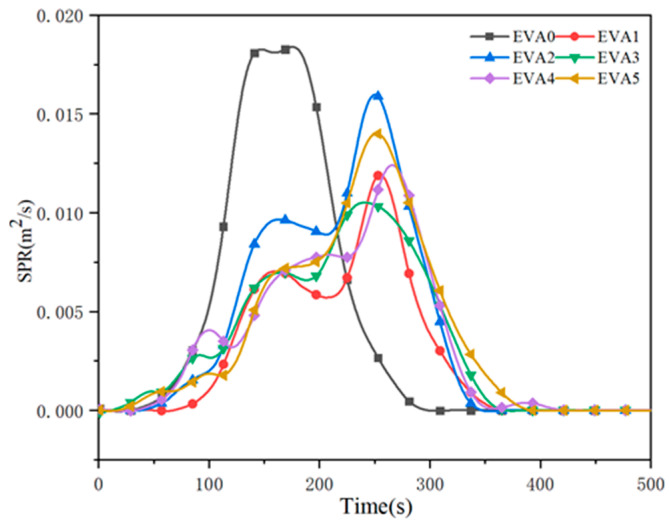
Smoke production rate curves of neat EVA and EVA/MH composites.

In a fire scenario, smoke and toxic gases are regarded as critical factors concerning human survival [28,29]. The smoke production rate (SPR) values of neat EVA and EVA/MH composites are illustrated in Figure 10. It was observed that neat EVA displayed a high peak SPR value of 0.018 m^2^/s. With the addition of 50 wt% MH, the peak SPR value of EVA1 dramatically decreased to 0.012 m^2^/s, corresponding to a 33% reduction. Similarly, the peak SPR values of EVA2, EVA3, EVA4 and EVA5 composites decreased from 0.018 m^2^/s for EVA0 to 0.016, 0.012, 0.013 and 0.014 m^2^/s, respectively. Typically, two peaks appeared in all of the SPR curves of the composites, which indicates the generation and damage of the carbonaceous layer. The emergence time of the peaks was delayed simultaneously. The formation of the two peaks could be due to the following: the samples released a large amount of combustion products after being ignited, leading to a sharp rise in SPR. When a layer of carbon formed during combustion, the value of SPR dropped after the first peak. When the samples were further exposed to heat, the protective layer was burned through, causing the increasing release of combustible gases. The SPR curves rose to a second peak until the samples were burned out [30].

**Figure 11:**
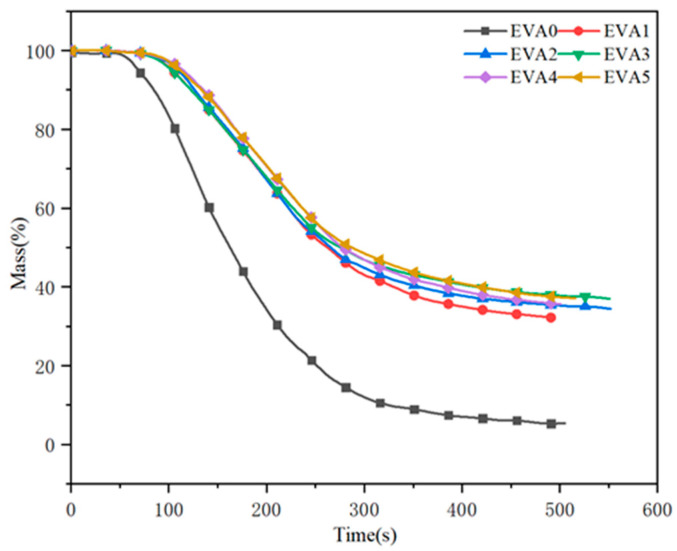
Mass curves of neat EVA and EVA/MH composites.

Fire resistance can also be evaluated from the residues present after the combustion of the materials [31]. The dynamic mass loss versus time curves for neat EVA and EVA/MH composites are recorded in Figure 11. It can be seen that neat EVA lost its mass faster than the specimens with MH, with only 5.3 wt% char residue remaining at 500 s. In detail, the char residues of EVA1 to EVA5 were 32.3%, 34.6%, 37.0%, 35.4%, 37.2%, respectively. Regarding the EVA5 sample, the char residue was highest among all of the composites, and the value of EVA3 was less than that of EVA5. During the combustion process, a compact char might grow on the surface of the burning sample.

**Figure 12:**
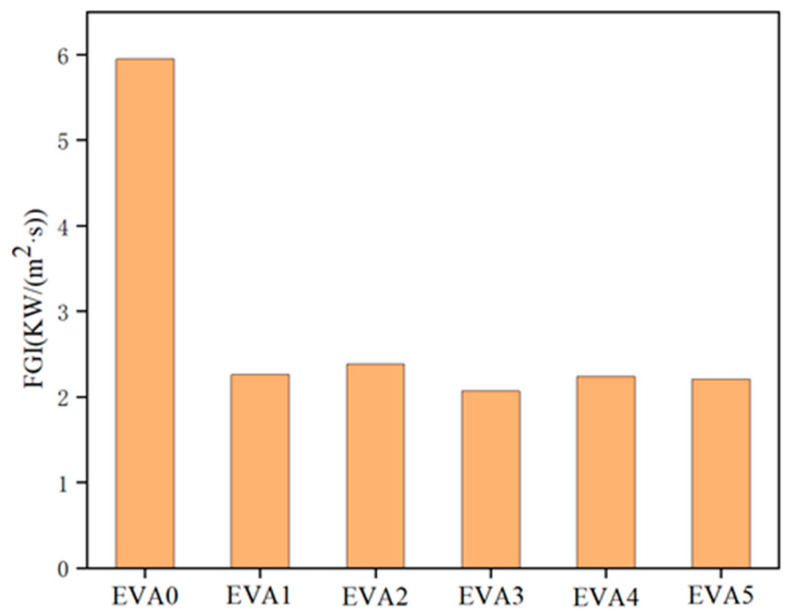
Fire growth index for neat EVA and EVA/MH composites.

In parallel, the fire safety performance of materials is analyzed via the fire growth index (FGI) [32]. The values of FGI of the specimens are shown in Figure 12 and Table 5. As expected, the FGI markedly decreased after adding MH, indicating that MH could control the spread of fire and reduce the fire intensity. The EVA3 sample had the lowest FGI, indicating a lower fire risk.

### 3.6. Carbon Residue Analysis

**Figure 13:**
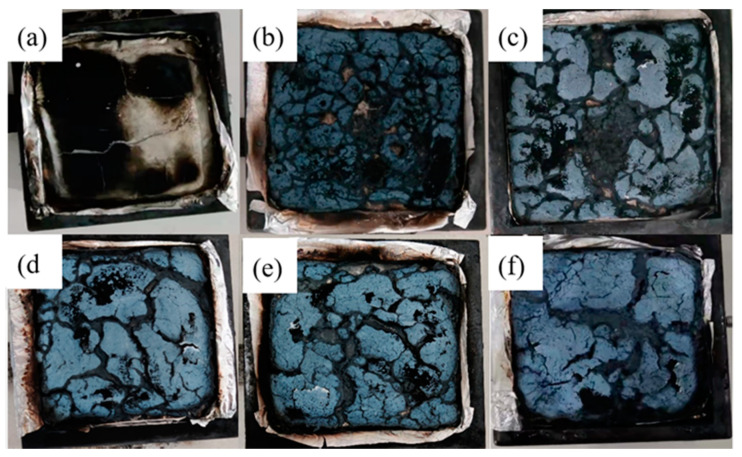
Digital photos of the charred residue samples collected after cone calorimeter tests. (**a**) EVA0; (**b**) EVA1; (**c**) EVA2; (**d**) EVA3; (**e**) EVA4; (**f**) EVA5.

Figure 13 shows the macro-morphologies of the final chars after CCT of the samples. It can be seen that EVA0 left few charred residues, which is in agreement with the previous studies of mass loss curves. Obvious holes and cracks can be observed in the char residues of EVA1. More smoke and harmful gas produced by the combustion of composites can be released through cracks. Furthermore, the char structures of EVA3 and EVA5 were noticeably more continuous and compact than other composites, thereby resulting in a higher residue, which is regarded as a better carbonaceous layer structure. The results showed that the char structure of the composites was more complete when MH prepared in an alkaline environment was added to EVA, isolating oxygen and heat from materials, inhibiting the volatilization of smoke and improving the performance of the flame retardant. The results of combustion photos are consistent with the HRR and SPR values of composite materials.

### 3.7. Mechanical Properties

**Figure 14:**
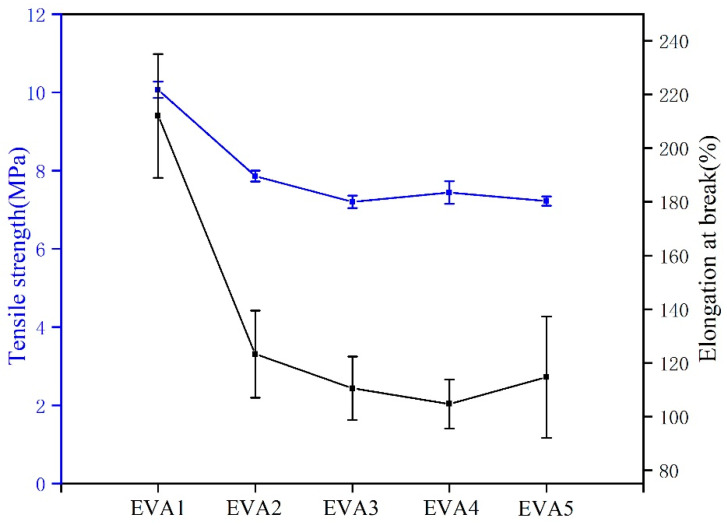
The mechanical properties of EVA/MH composites.

The mechanical properties of EVA/MH composites were characterized by the tensile test. The tensile strength and elongation at break data of EVA/MH composites are shown in Figure 14. Compared with EVA1, a slight decrease in tensile strength from 10.0 MPa to around 8.0 MPa for EVA2, EVA3, EVA4 and EVA5 can be observed. On the other hand, MH synthesized in an alkaline environment showed a stronger impact on the elongation at break of EVA/MH composites. The results indicated that the introduction of MH led to the deterioration of the mechanical properties of polymer materials [33]. Nevertheless, the resulting composites could still meet the requirements of wire and cable sheath material [30].

## 4. Conclusions

In this work, hydromagnesite was used to synthesize magnesium hydroxide (MH) as a flame retardant for EVA. Various concentrations of sodium hydroxide (NaOH) were used to alter the morphology and the flame-retardant efficiency of synthesized MH. The influence of NaOH on the flame retardancy and mechanical properties was investigated. The LOI values of samples drastically increased after introducing MH into EVA. EVA4 showed an LOI result as high as 37.9%. The CCT test results showed that the pHRR, THR and the peak of SPR of EVA3 were decreased by 37.6%, 20.7% and 44.4% compared with neat EVA. The digital photos confirmed that compact char residues were formed for EVA3 and EVA5 after combustion. In sum, MH prepared in an alkaline environment was much more efficient in enhancing the flame retardancy of EVA. At the same time, the tensile strength of all specimens exceeded 7.0 MPa. These composites can meet the requirements of wire and cable sheath material.

## Figures and Tables

**Table 1:** Formulations of neat EVA and EVA/MH composites.

Sample	EVA (wt%)	MH (wt%)	NaOH Concentration (mol/L)
EVA0	100	0	-
EVA1	50	50	0
EVA2	50	50	0.5
EVA3	50	50	1.0
EVA4	50	50	1.5
EVA5	50	50	2.0

**Table 2:** TG data of hydromagnesite sample under nitrogen.

Sample	T_−5%_ (°C)	T_−50%_ (°C)	T_max1_ (°C)	T_max2_ (°C)	T_max3_ (°C)	Residue at 800 °C (wt%)
Hydromagnesite	233	481	240	450	618	52.82

**Table 3:** I_(001)_/I_(101)_ and mean particle sizes of MH powders.

NaOH Concentration/mol·L^−1^	0	0.5	1.0	1.5	2.0
I_(001)_/I_(101)_	1.05	1.08	1.21	1.32	1.18
Mean particle sizes/μm	2.60	3.45	3.66	3.72	3.93

**Table 4:** TG data of neat EVA and EVA/MH composites.

Samples	T_−5%_^a^ (°C)	T_max1_^b^ (°C)	T_max2_ ^c^ (°C)	Residue ^d^ (wt%)
EVA0	331.8	348.9	459.9	0
EVA1	336.1	353.5	460.7	35.0
EVA2	333.7	348.0	462.1	35.4
EVA3	333.2	352.1	462.4	35.5
EVA4	333.4	349.7	460.2	35.8
EVA5	334.2	354.7	462.8	35.6

^a^ Temperature at 5 wt% weight loss. ^b^ Temperature at first maximum mass loss rate. ^c^ Temperature at second maximum mass loss rate. ^d^ Residue at 800 °C.

**Table 5:** Cone calorimetric data of neat EVA and EVA/MH composites.

Samples	TTI ^a^ (s)	pHRR ^b^ (kW/m^2^)	TpHRR ^c^ (s)	THR ^d^ (MJ/m^2^)	TSP ^e^ (m^2^)	FGI ^f^ (KW/(m^2^·s))	Residue ^g^ (wt%)
EVA0	50	559	100	82	4.9	5.95	5.3
EVA1	83	381	169	65	1.4	2.26	32.3
EVA2	82	374	157	74	5.3	2.38	34.6
EVA3	77	349	168	65	2.9	2.07	37.0
EVA4	94	378	169	68	6.7	2.24	35.4
EVA5	76	359	169	70	8.5	2.21	37.2

^a^ TTI, time to ignition. ^b^ pHRR, peak of heat release rate. ^c^ TpHRR, time of pHRR. ^d^ THR, total heat release. ^e^ TSP, total smoke production. ^f^ FGI, pHRR/TpHRR. ^g^ Residue, mass percentage of carbon residue when flame is extinguished.

## Data Availability

The data presented in this study are available on request from the corresponding author.
